# Liqi Yangyin formula ameliorates CUMS-induced depression and comorbid constipation via ACE/FFAR2 modulation of the microbiota-gut-brain axis

**DOI:** 10.3389/fcimb.2025.1692110

**Published:** 2025-11-07

**Authors:** Lianjie Xu, Shun Seng Ong, Xiaoyue Deng, Yunzhi Qian, Hai Lu, Yang Song, Yang Wang, Tianshu Xu

**Affiliations:** 1 Department of Traditional Chinese Medicine, Nanjing Drum Tower Hospital, The Drum Tower Clinical Medical College, Nanjing University of Chinese Medicine, Nanjing, China; 2 Department of Nutrition, University of North Carolina at Chapel Hill, Chapel Hill, NC, United States; 3 Department of Traditional Chinese Medicine, Nanjing Drum Tower Hospital, Affiliated Hospital of Medical School, Nanjing University, Nanjing, China

**Keywords:** depression, constipation, the gut-brain axis, neuroinflammation, ACE/FFAR2, 5-HT

## Abstract

**Background:**

The gut-brain axis, involving bidirectional signaling between the gastrointestinal tract and the central nervous system. Clinical observations have shown that Liqi Yangyin (LQYY) can effectively relieve symptoms of depression accompanied by constipation. However, whether LQYY exerts its effects through gut-brain crosstalk remains to be elucidated.

**Methods:**

A chronic unpredictable mild stress (CUMS) protocol was employed to establish a mouse model. H&E and Nissl staining were used to examine pathological changes in the prefrontal cortex (PFC) and colon. The ultrastructure of the intestinal barrier was observed via transmission electron microscopy, while the expression of the blood-brain barrier tight junction proteins was quantified by Western blotting (WB). ELISA quantified inflammatory factors and serotonin (5-HT) levels. Immunohistochemistry, immunofluorescence, and WB analyzed IBA-1 and Free fatty acid receptor 2 (FFAR2) expression levels. Gut microbiota composition was analyzed via 16S rDNA sequencing, and SCFAs levels were quantified using UHPLC-TSQ Altis Plus. Additionally, *in vitro* studies using BV-2 cells involved treatments with acetic acid (ACE) and an FFAR2 antagonist, after which the expression of relevant indicators was assessed.

**Results:**

Our results demonstrated that LQYY significantly ameliorated CUMS-induced behavioral changes and improved intestinal motility. These effects were associated with the restoration of gut microbiota balance and an increase in ACE levels. LQYY increased FFAR2 expression, leading to reduced neuroinflammation and enhanced colonic 5-HT secretion. Furthermore, LQYY protected intestinal and blood-brain barrier integrity and improved neuronal morphology in the PFC. *In vitro* studies confirmed that ACE suppresses microglial inflammation through upregulating FFAR2 expression, an effect that was attenuated by the FFAR2 inhibitor GLPG0974.

**Conclusions:**

These findings suggest that LQYY modulates the gut-brain axis through ACE/FFAR2, offering a promising therapeutic approach for depression and constipation.

## 1 Introduction

Depression, a mental disorder defined by prolonged sadness or diminished interest, often persists for two weeks or longer. In severe cases, individuals may exhibit suicidal ideation, alongside common prodromal symptoms such as anxiety, irritability, tension, and somatic discomfort (Benasi et al., 2021). Global statistics indicate that over 350 million people are affected by depression, with the condition’s prevalence increasing yearly (Zeng et al., 2023). Constipation is an intestinal disorder marked by symptoms like reduced bowel frequency, difficulty passing stools, incomplete bowel emptying, or the passage of dry, hard feces. This condition is prevalent worldwide, with a reported global prevalence of approximately 15% (Feng et al., 2024). Depression and constipation often coexist in medical settings, with a substantial number of patients experiencing both conditions at the same time (Ballou et al., 2019). A Mendelian randomization analysis was carried out to investigate the causal link between depression and constipation. The findings demonstrated a significant causal association between the two conditions, suggesting that depression could lead to the development of constipation. Furthermore, the intensity of constipation symptoms was associated with the severity of depressive symptoms (Wu et al., 2024). Moreover, animal experiments have confirmed that depressive mouse models exhibit intestinal motility disorders (Israelyan et al., 2019). Recent studies have demonstrated that CUMS-induced depression in mice results in a series of constipation-related symptoms, such as reduced intestinal movement and fecal water content (Chen et al., 2025). This finding underscores the key role of the gut-brain axis in linking emotional disturbances with gastrointestinal function. The gut-brain axis enables two-way signaling between the central nervous system (CNS) and the gastrointestinal tract, involving neurological, endocrine, and immune mechanisms (Cryan et al., 2019).

The brain can regulate intestinal function through immune responses, thereby influencing the gut microbiota and potentially leading to constipation. Conversely, when gut microbiota dysbiosis occurs, intestinal metabolites can affect the brain via the gut-brain axis, contributing to the development of depression (Margolis et al., 2021). In recent years, growing evidence has shown that chronic stress can induce gut microbiota dysbiosis (Chang et al., 2022) and trigger morphological and functional changes in microglia, contributing to the development of depression (Frank et al., 2019). SCFAs, the main products of gut microbiota, mainly consist of ACE, propionic acid, and butyric acid. These molecules can cross the blood-brain barrier (BBB), exhibit anti-inflammatory properties, and are implicated in maintaining microglial homeostasis (Li et al., 2022a). Mechanistically, SCFAs regulate immune and inflammatory responses by activating receptors such as FFAR2, also referred to as G protein-coupled receptor 43 (GPR43) (Li et al., 2018). FFAR2 is present in the intestine, brain, and adipose tissue and contributes to the development and function of immune cells within the brain (Cheng et al., 2024; Ikeda et al., 2022). Notably, SCFAs have been shown to enhance 5-HT release by increasing FFAR2 expression on enterochromaffin cells (ECs), thereby alleviating constipation (Reigstad et al., 2015; Iwasaki et al., 2019; Tuohongerbieke et al., 2024). However, the potential of SCFAs to inhibit microglial activation via FFAR2 and suppress neuroinflammation for depression treatment remains to be explored.

In clinical practice, Western medicine typically employs symptomatic treatment for depression accompanied by constipation. However, commonly used antidepressants often cause gastrointestinal adverse reactions, such as nausea, vomiting, diarrhea, and constipation (Oliva et al., 2021). Additionally, long-term use of medications such as osmotic laxatives and prokinetic agents can result in adverse effects, including medication tolerance and reduced colonic motility (Wu et al., 2025). Thus, identifying approaches to alleviate depressive symptoms while reducing constipation and its associated side effects is of significant importance. LQYY, a traditional Chinese medicine, is recognized for its capacity to regulate qi, relieve depression, nourish yin, and promote defecation. We have previously identified the components in the LQYY formula by LC-MS and screened with an abundance ≥1%, including citric acid, DL-Stachydrine, adenine, naringin, hesperidin, and D-quinic acid. And it has also proven that it can restore gut microbiota balance, increases SCFAs levels, stimulates ECs to secrete 5-HT, enhances intestinal motility, and alleviates constipation (Li et al., 2024). Among them, the active ingredients such as naringin and hesperidin not only have antidepressant effects, but also can increase the abundance of beneficial bacteria, inhibit the harmful microorganisms, and maintain the intestinal balance, thereby conferring certain benefits to host health (Gao et al., 2022; Huang et al., 2025; Emran et al., 2022; Fu et al., 2019; Lee et al., 2021). Clinical observations have shown that LQYY can effectively relieve symptoms of depression accompanied by constipation. However, whether LQYY exerts its effects through gut-brain crosstalk remains to be elucidated.

In this study, we hypothesize that LQYY may alleviate behavioral changes and constipation symptoms in a CUMS-induced mouse model. We propose that the therapeutic benefits of LQYY could be attributed to the restoration of gut microbiota balance, an increase in ACE and FFAR2 levels, inhibition of microglial inflammation in the PFC, and enhancement of colonic 5-HT secretion.

## 2 Materials and methods

### 2.1 Animals and interventions

Seven-week-old SPF male C57BL/6 mice were purchased from Jiangsu Huachuang Xinnuo Pharmaceutical Science and Technology Co., Ltd. and housed under controlled conditions (23 ± 2°C, 60% humidity, and 12/12 h light/dark cycles). All procedures complied with institutional guidelines and were approved by the Laboratory Animal Ethics Committee of Nanjing Drum Tower Hospital, Nanjing University Medical School (No. 2024AE01019).

Behavioral change was induced by CUMS as described previously (Chen et al., 2025): for six weeks, mice experienced two randomly selected stressors per day drawn from food and water deprivation (24 h), 45°cage tilt (24 h), empty-cage exposure (24 h), horizontal shock administered at one per second (10 min), swimming in 40 °C water (10 min), light–dark cycle reversal (24 h), 2 cm from the tip of tail pinching (5 min), wet bedding (24 h), and bodily restraint (3 h). After a 7-day acclimation, animals were randomized to five groups (n = 8/group): Control, CUMS, LQYY, mosapride citrate (MC), and fluoxetine (FLX). Except for Controls, all groups underwent CUMS for six weeks and then received once-daily oral treatment for four weeks: LQYY (15, 30, or 60 g/kg/day), MC (3 mg/kg/day; H19990313; Chengdu Kanghong Pharmaceutical Group Co., Ltd.; Zhou et al., 2024), or FLX (10 mg/kg/day; HJ20181215; Patheon France; Chen et al., 2025). Body weight was recorded weekly.

Following behavioral testing, including the sucrose preference test (SPT), open field test (OFT), forced swimming test (FST), and novelty-suppressed feeding test (NSFT), the time to first black stool was recorded, and fecal water content was measured. Fresh fecal samples were collected aseptically and stored at −80 °C. Mice were euthanized by cervical dislocation; blood was kept at 4 °C, centrifuged at 2,500 rpm for 15 min, and serum stored at −80 °C. Segments of colon and PFC were fixed in 4% paraformaldehyde for histology; additional colon was placed in electron-microscopy fixative for transmission electron microscopy, and remaining PFC and colon were snap-frozen at −80 °C for subsequent analyses.

In a separate microbiota-depletion cohort (ABX), mice were randomized to five groups (n = 8/group), and—prior to LQYY administration—a broad-spectrum antibiotic cocktail was administered to the mice via their drinking water, and the subsequent impact of this intervention on the effects of LQYY was then assessed: ampicillin (1 g/L; A830931; Macklin), vancomycin (0.5 g/L; V820413; Macklin), neomycin sulfate (1 g/L; N8090; Solarbio), and metronidazole (1 g/L; M813526; Macklin) (Hao et al., 2024).

### 2.2 Composition of LQYY formula

LQYY formula were acquired from Nanjing Drum Tower Hospital, it is composed of 12 distinct Chinese herbs: *Adenophora triphylla* (Thunb.) A.DC. (Nan Sha Shen, 20g), *Ophiopogon japonicus* (Thunb.) Ker Gawl. (Mai Dong, 20g), *Scrophularia ningpoensis* Hemsl. (Xuan Shen, 30g), *Rehmannia glutinosa* (Gaertn.) Libosch. ex DC. (Shu Di Huang, 30g), *Prunus armeniaca* L. (Xing Ren, 10g), *Atractylodes macrocephala* Koidz. (Bai Zhu, 40g), *Citrus × aurantium* L. (Zhi Qiao, 15g), *Magnolia officinalis* Rehder & E.H.Wilson (Hou Po, 10g), *Trichosanthes kirilowii* Maxim. (Gua Lou Ren, 20g), *Cannabis sativa* L. (Huo Ma Ren, 10g), *Prunus humilis* Bunge (Yu Li Ren, 15g), and *Dolomiaea costus* (Falc.) Kasana & A.K.Pandey (Mu Xiang, 8g), with botanical names verified via MPNS (https://mpns.kew.org). The total crude weight was 228 g. The mixture was soaked in water for 45 min, decocted twice, and the combined filtrates were concentrated to 3 g/mL, then sterilized by high-temperature autoclaving for subsequent use.

### 2.3 BV-2 cells culture and treatment

The CCK-8 assay was performed on BV-2 cells (cat. no.CL-0493, Wuhan Pricella Biotechnology Co., Ltd.) to determine the optimal drug concentration based on cell viability. Cells were maintained in culture flasks with DMEM (G4515, Servicebio) supplemented with 10% fetal bovine serum (A5256701, Gibco) and 1% penicillin-streptomycin (G4003, Servicebio) at 37 °C in 5% CO_2_. For viability testing, 1 × 10^4^ cells per well were seeded into 96-well plates, allowed to reach ~80-90% confluence, and then exposed for 24 h to lipopolysaccharide (LPS; L6529, Sigma) at 0.1, 0.5, 1, or 2 µg/mL, the FFAR2 antagonist GLPG0974 (1391076-61-1, Proteintech) at 1, 5, 10, or 20 µM, or sodium acetate (ACE; S5636, Sigma) at 10, 20, or 40 mM. Next, 10 µL CCK-8 reagent (C0037, Beyotime) was added to each well and plates were incubated a further 1–4 h at 37 °C/5% CO_2_ before measuring absorbance at 450 nm on a SPARK multimode reader. For downstream assays, BV-2 cells were plated in 96- and 6-well plates at appropriate densities and, upon reaching ~90% confluence, assigned to five groups: Control, LPS, LPS + ACE, LPS + ACE + GLPG, and LPS + GLPG. Where indicated, cells were pretreated with GLPG0974 (10 µM) for 12 h, followed by stimulation with LPS (1 µg/mL) with or without ACE (20 mM) for 12 h; enzyme-linked immunosorbent assay (ELISA), WB, and immunofluorescence (IF) were then performed as described.

### 2.4 Behavioral tests

#### 2.4.1 Sucrose preference test

Mice were first habituated by providing two bottles of 1% sucrose for 24 h, followed by a 24 h training phase with one bottle of 1% sucrose and one bottle of water, with bottle positions alternated randomly. After 24 h of food and water deprivation, the formal test began: mice received one bottle of 1% sucrose and one bottle of water for 12 h, with positions switched every 6 h. Sucrose (C1) and water (C2) intake were recorded, and sucrose preference was calculated as SCR (%) = C1/(C1 + C2) × 100.

#### 2.4.2 Open field test

Each mouse was placed in a square arena (45 × 45 × 45 cm) for a 10 min acclimation, then returned to the same start position for a 5 min recording session. Movements were tracked with an automated video system (TopScan v3.0, CleverSys, USA). Primary outcomes were time spent in the central area and total path length. The arena was cleaned with 75% ethanol between trials to eliminate residual odors.

#### 2.4.3 Forced swimming test

The FST was performed as described previously (Zhou et al., 2024). Each mouse was placed individually in a transparent cylinder (25 cm height, 10 cm diameter) containing 20 cm of water at 25 ± 1 °C and allowed to swim for 6 min in a quiet room while behavior was video-recorded. Immobility—defined as the absence of active struggling with only minimal movements to keep the head above water—was quantified with a stopwatch during the final 4 min.

#### 2.4.4 Novelty-suppressed feeding test

The NSFT was performed as described previously with minor modifications (Zhang et al., 2023). Each mouse was individually habituated to a black open box for 10 min and then returned to its home cage. After 24 h of food deprivation, a food pellet was placed at the center of the same arena and the mouse was reintroduced for a 5 min test; the latency to the first bite (feeding latency) was recorded from test onset. Between trials, the object and arena were cleaned with 75% ethanol to remove residual odors.

### 2.5 Intestinal function tests

#### 2.5.1 Fecal water content

After testing, fresh fecal pellets were collected into pre-weighed 2 mL microcentrifuge tubes. The wet mass (W1) was obtained by subtracting the empty-tube weight from the tube plus moist feces. Samples were dried for 3 h at 100 °C in a thermostatic oven, and the dry mass (W2) was determined similarly. Fecal water content (FWC, %) was calculated as FWC = (W1 − W2)/W1 × 100.

#### 2.5.2 First black stool

The charcoal suspension was prepared by dissolving 1 g gum acacia in 80 mL distilled water and boiling until clear. After cooling slightly, 5 g activated carbon was added, and the mixture was brought to a boil three times to ensure homogeneity. Once cooled to room temperature, the suspension was adjusted to 100 mL with distilled water. Mice were fasted for 24 h with free access to water before testing. The charcoal suspension was mixed thoroughly and administered by oral gavage at 0.1 mL per 10 g body weight. Each mouse was then housed individually in a clean cage for observation. The time of gavage (T1) and the time of first black stool (T2) were recorded, and the latency to first black stool was calculated as T2 − T1.

#### 2.5.3 Intestinal propulsion rate

Mice were fasted for 12 h with water available ad libitum, then gavaged with a 5% activated-carbon suspension. Thirty minutes after gavage, the entire intestine—from the pylorus to the anal verge—was excised in one piece, the total length (L1) and the distance traveled by the carbon front (L2) were measured, and the intestinal propulsion rate (IPR, %) was calculated as IPR = (L2/L1) × 100.

### 2.6 Bioassays

#### 2.6.1 Histopathological testing of PFC and colon

Mice PFC and colonic tissues were preserved in 4% paraformaldehyde for 48 h. After the fixation process, the tissues were embedded in paraffin and sectioned into 4 μm thick slices. The histological morphology of PFC and colonic tissue sections were examined using H&E staining. Additionally, the structural integrity of neurons in the PFC was observed using Nissl staining. The stained sections were analyzed using a Zeiss MDS brightfield scanning microscope.

#### 2.6.2 Colon ultrastructural morphology

Fresh colonic tissue samples (1 mm³) were harvested and promptly fixed in electron microscopy fixative at 4 °C overnight. The samples were then washed in a 0.1 M phosphate buffer, followed by sequential ethanol dehydration, embedding, sectioning, staining, and drying. The ultrastructure of the colonic tissue was examined via transmission electron microscopy (HT7800; Hitachi).

#### 2.6.3 ELISA testing of PFC, colon, serum and cell supernatant

ELISA kits (Elabclone, Nanjing, China) were used according to the manufacturer’s instructions to quantify TNF-α, IL-6, 5-HT, and BDNF in PFC tissues; TNF-α, IL-6, and 5-HT in colonic tissues; and TNF-α and IL-6 in serum and cell supernatant. Absorbance at 450 nm was measured on a SPARK multimode reader (Tecan), and analyte concentrations were calculated.

#### 2.6.4 Immunofluorescence staining of PFC, colon and BV-2 cells

PFC and colonic tissues were sectioned at 4 μm, deparaffinized in xylene, and rehydrated through graded ethanol to water. Antigen retrieval was performed in citrate buffer (pH 6.0) followed by PBS washes. Endogenous peroxidase was quenched with 3% H_2_O_2_ for 15 min at room temperature. PFC sections were incubated with primary antibodies to IBA-1 (1:200, ab283319, Abcam) and FFAR2 (1:100, 19952-1-AP, Proteintech), then with fluorescent secondary antibodies (555 goat anti-mouse and 488 goat anti-rabbit). Colonic sections were incubated with anti-FFAR2 (1:100) followed by 488 goat anti-rabbit. For cells, round coverslips placed in 6-well plates were seeded with BV-2 cells (1 × 10^6^ per well). After drug treatment, cells were fixed in 4% paraformaldehyde for 20 min, permeabilized with 0.5% Triton X-100 for 20 min, blocked with 3% bovine serum albumin, and incubated overnight at 4 °C with primary antibodies against IBA-1 (1:100) and FFAR2 (1:200). After washing, appropriate fluorophore-conjugated secondary antibodies were applied. Nuclei were counterstained with DAPI, an antifade mounting medium was applied, and images were acquired on a THUNDER system (Leica Microsystems, Germany).

#### 2.6.5 Western blotting of PFC and BV-2 cells

PFC tissues and BV-2 cells were lysed in RIPA buffer (Servicebio, China), and protein concentrations were determined using a BCA assay kit (Servicebio, China). Equal amounts of protein were resolved on 10% SDS-PAGE precast gels (PAGE Color Gel Ultra Rapid Preparation Kit; Servicebio, China) and transferred to PVDF membranes. Membranes were blocked with 5% skim milk for 2 h at room temperature and incubated overnight at 4°C with primary antibodies against ZO-1 (21773-1-AP, Proteintech), claudin-5 (66879T, CST), occludin (DF7504, Affinity), and FFAR2. After washing three times with TBST, the membranes were incubated with secondary antibodies for 1 h at room temperature. Proteins were identified via an enhanced chemiluminescence (ECL) reagent. The intensities of the protein bands were quantified through the ImageJ software.

#### 2.6.6 Immunohistochemistry of PFC

PFC tissues were sectioned at 4 μm. Sections were deparaffinized in xylene, rehydrated through graded ethanol to water, and subjected to antigen retrieval in citrate buffer (pH 6.0). After PBS rinses, endogenous peroxidase was quenched with 3% H_2_O_2_ for 15 min. Slides were incubated overnight at 4 °C with primary antibodies against IBA-1 (1:500) and FFAR2 (1:200), followed by HRP-conjugated secondary antibodies. They were stained with 3,3′-diaminobenzidine (DAB), nuclei were counterstained with hematoxylin, and images were acquired using a Zeiss MDS brightfield scanning microscope.

### 2.7 16S rDNA sequencing and analyses

Total microbial DNA was extracted from fecal samples using the PF Mag-Bind Stool DNA Kit (Omega Bio-tek, GA, USA) according to the manufacturer’s instructions, and DNA integrity and yield were assessed by 1.0% agarose gel electrophoresis. The V3-V4 hypervariable region of the bacterial 16S rDNA gene was amplified with universal primers 338F (5′-ACTCCTACGGGAGGCAGCAG-3′) and 806R (5′-GGACTACHVGGGTWTCTAAT-3′). Amplicons were excised from 2% agarose, purified, and quantified on a Quantus™ fluorometer (Promega, USA). Libraries were sequenced on an Illumina platform, and downstream analyses were performed using the SILVA database for taxonomic annotation and abundance profiling. Sequencing and bioinformatics were conducted by Hangzhou Cosmos Wisdom Biotechnology Co., Ltd. (Hangzhou, China).

### 2.8 Detection of SFCAs in feces

Fecal SCFAs were quantified by UHPLC-TSQ Altis Plus. Briefly, 20 mg of feces was homogenized in 1,000 μL of 80% methanol by vortexing and mechanical disruption, then centrifuged. A 30 μL aliquot of the supernatant was combined with 10 μL internal standard solution and derivatized at 30 °C for 30 min. After a second centrifugation, the clarified supernatant was subjected to analysis.

### 2.9 Statistical analysis

Results were analyzed with SPSS version 25.0 (IBM Corp.) and GraphPad Prism version 9.5 software (Graphpad Software Inc.). Data are presented as the mean ± SD from at least three independent experiments. Differences between multiple groups were determined using one-way ANOVA with Tukey’s *post hoc* test after being assessed with the Shapiro-Wilk test for normal distribution. In cases where data deviated from a normal distribution or exhibited heterogeneous variance, the non-parametric Kruskal-Wallis test for K-independent samples was applied. A *p*-value of less than 0.05 was considered statistically significant.

## 3 Results

### 3.1 LQYY alleviates CUMS-induced depression and constipation in mice

**Figure 1:**
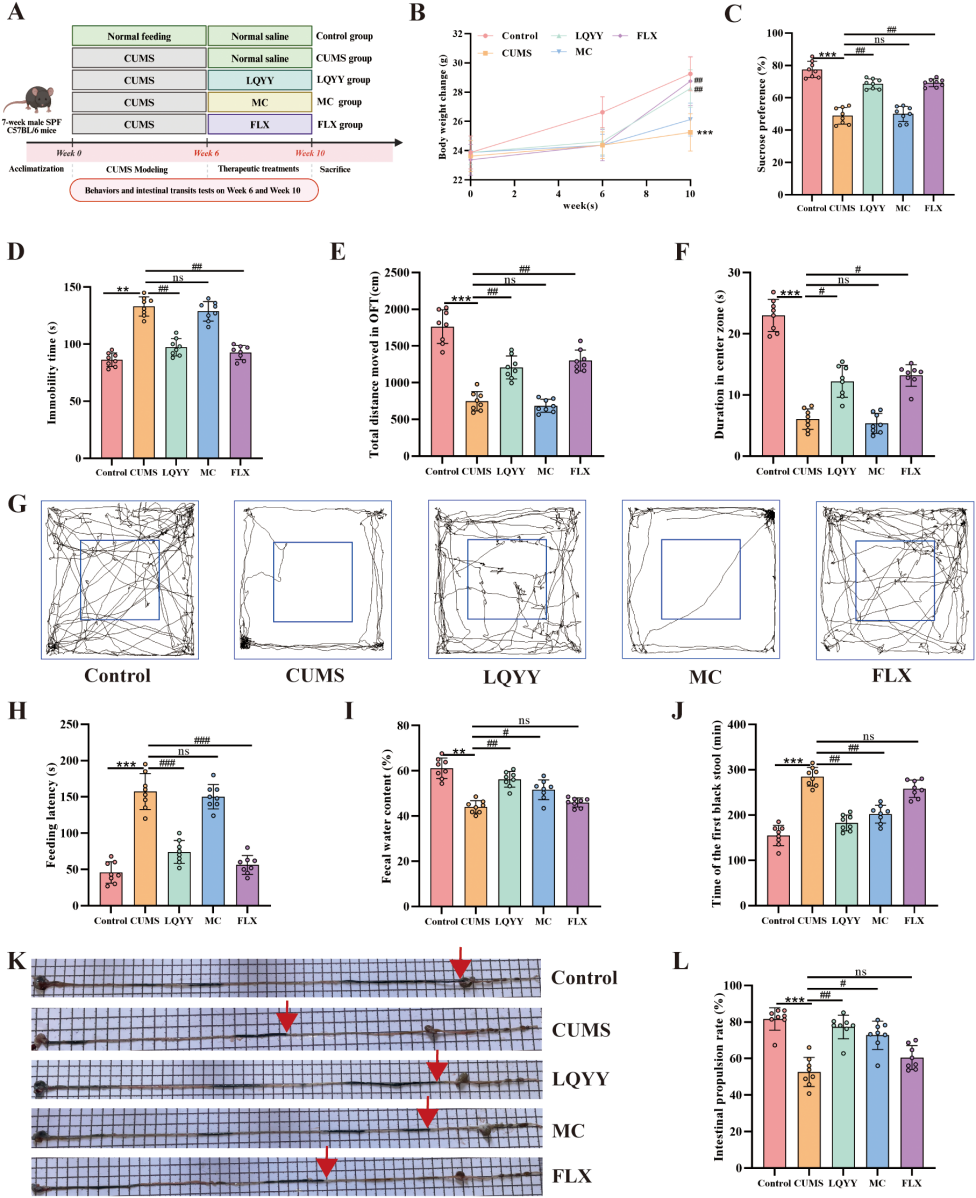
LQYY ameliorates CUMS-induced behavioral changes and intestinal transit disorders. **(A)** Experimental timeline. **(B)** Body weight across the study. **(C)** SPT. **(D)** FST immobility time. **(E)** OFT total distance. **(F)** Open-field center time. **(G)** Open-field movement trajectories. **(H)** NSFT feeding latency. **(I)** Fecal water content. **(J)** Time of the first black stool. **(K)** Representative image of activated-carbon transit; red arrow marks the carbon front. **(L)** Intestinal propulsion rate. (n = 8 per group). **P < 0.01, ***P < 0.001 *vs* control; ^#^*P* < 0.05, ^##^*P* < 0.01, ^###^*P* < 0.001 *vs* CUMS.

Following the 6-week CUMS paradigm (**Figure 1A**), a dose of 30 g/kg/day LQYY was selected for subsequent experiments based on a preliminary dose-finding analysis (**Supplementary Figure S1**). Compared to the CUMS group, LQYY-treated mice showed increased body weight gain, higher sucrose preference in the SPT, greater total distance and center time in the OFT, reduced immobility time in the FST, and shorter latency to feed in the NSFT (**Figures 1B–H**). Regarding intestinal function, the LQYY group exhibited elevated fecal water content (**Figure 1I**), a shorter time to the first black stool (**Figure 1J**), and an increased intestinal propulsion rate (**Figures 1K, L**) relative to the CUMS group. In contrast, the antidepressant FLX did not significantly alter the measured parameters of intestinal transit, while the prokinetic agent MC did not reverse the CUMS-induced behavioral changes.

### 3.2 LQYY alleviates CUMS-induced depression and constipation in mice via gut microbiota-dependent mechanisms

**Figure 2:**
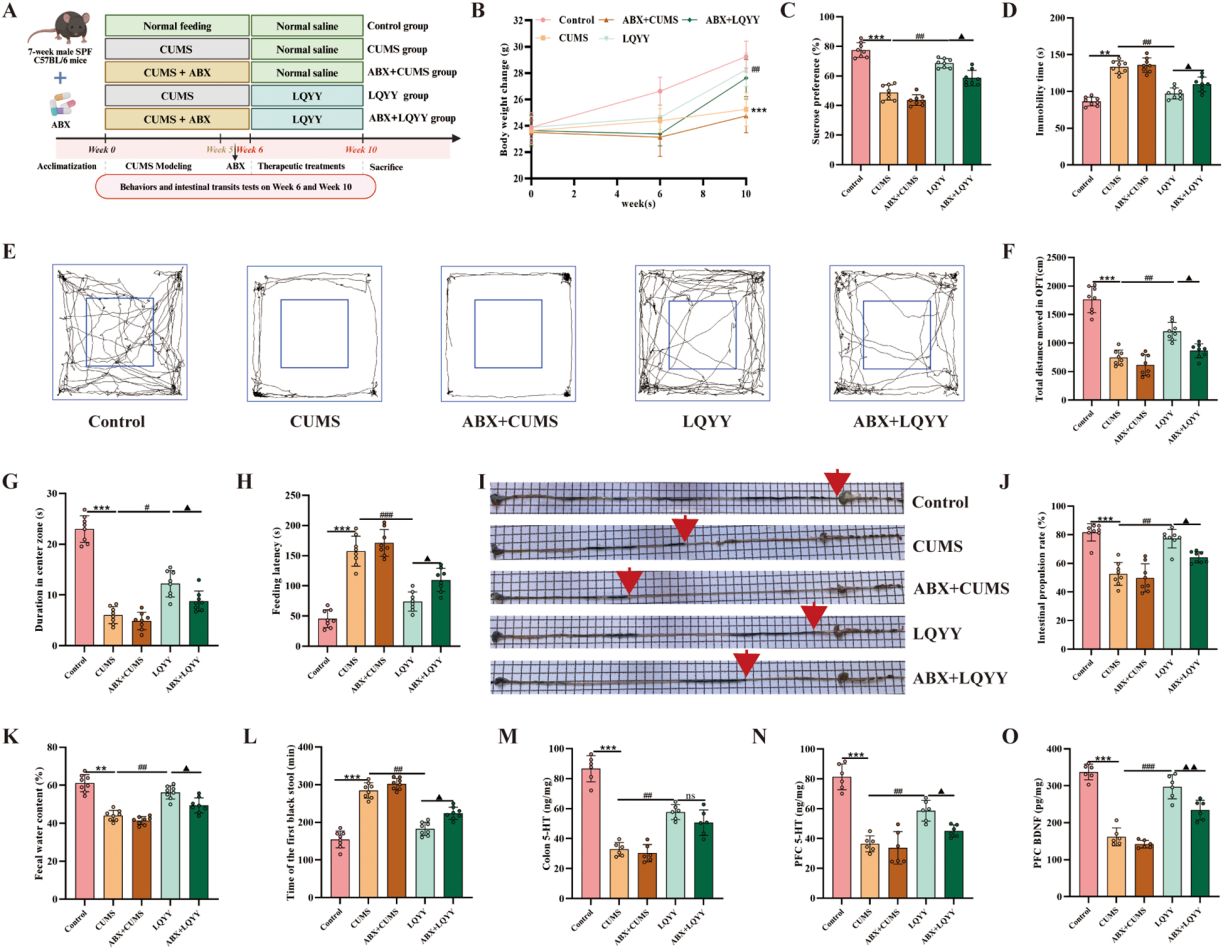
LQYY ameliorates CUMS-induced behavioral changes and intestinal transit deficits in a gut microbiota-dependent manner, and modulates neurotransmitters. **(A)** Experimental design. **(B)** Body weight across the study (n = 8). **(C)** SPT (n = 8). **(D)** The immobility time in FST (n = 8). **(E)** Movement trajectories in OFT (n = 8). **(F)** Total distance in OFT (n = 8). **(G)** The duration in center zone in OFT (n = 8). **(H)** The feeding latency in NSFT (n = 8). **(I, J)** Intestinal transit rate, the red arrow indicates the end of intestinal propulsion (n = 8). **(K)** Fecal water content (n = 8). **(L)** Time of the first black stool (n = 8). **(M)** Colonic 5-HT by ELISA (n = 6). **(N, O)** PFC 5-HT and BDNF by ELISA (n = 6). **P < 0.01, ***P < 0.001 *vs* control; ^#^*P* < 0.05, ^##^*P* < 0.01, ^###^*P* < 0.001 *vs* CUMS; ^▲^*P* < 0.05, ^▲▲^*P* < 0.01 *vs* LQYY.

Mice received an ABX in their drinking water for 1 week prior to the initiation of LQYY treatment (**Figure 2A**). In ABX-pretreated mice, the effects of LQYY on body weight gain were reduced (**Figure 2B**). The behavioral changes induced by LQYY, including those measured in the SPT, OFT, FST, and NSFT, were also attenuated in the ABX group (**Figures 2C–H**). Similarly, the improvements in intestinal transit parameters (fecal water content, time to first black stool, intestinal propulsion rate) following LQYY treatment were less pronounced in ABX-pretreated mice (**Figures 2I–L**). Consistently, ABX diminished LQYY-associated increases in colon 5-HT and in PFC 5-HT and BDNF (**Figures 2M–O**).

### 3.3 LQYY alleviates PFC damage and maintains BBB integrity in CUMS mice

**Figure 3:**
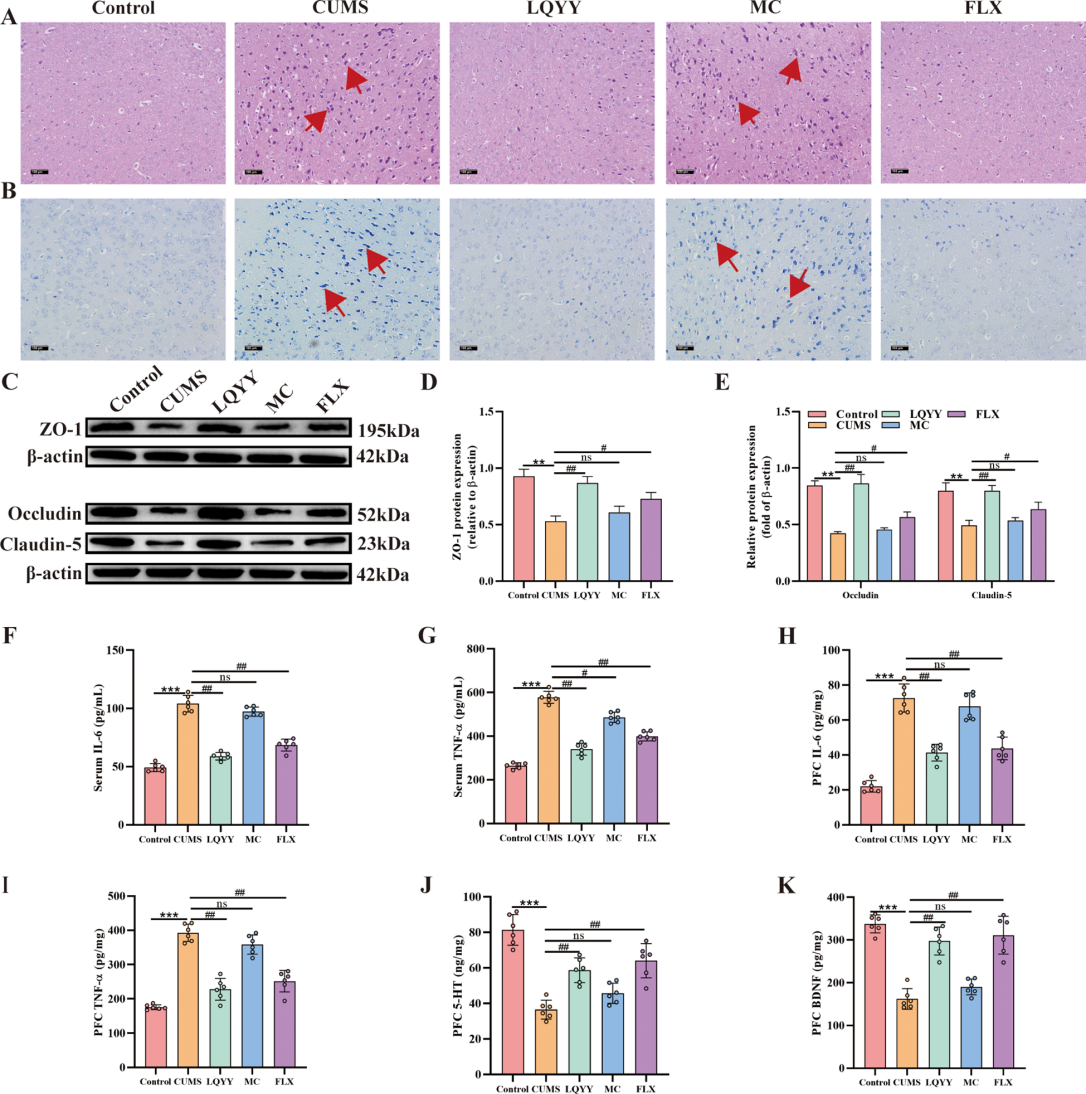
LQYY mitigates CUMS-induced PFC inflammation and BBB disruption. **(A, B)** H&E and Nissl staining of PFC (×200, scale bar = 100 μm), arrows indicate changes in cell morphology (n = 3). **(C-E)** The protein expression level of ZO-1, claudin-5 and occludin in PFC (n = 3). **(F, G)** Serum TNF-α and IL-6 by ELISA (n = 6). **(H, I)** PFC TNF-α and IL-6 by ELISA (n = 6). **(J, K)** PFC 5-HT and BDNF by ELISA (n = 6). **P < 0.01, ***P < 0.001 *vs* control; ^#^*P* < 0.05, ^##^*P* < 0.01, ^###^*P* < 0.001 *vs* CUMS.

In the PFC of CUMS mice, H&E staining revealed disorganized neuronal somata, nuclear pyknosis, and hyperchromasia compared to controls (**Figure 3A**). Nissl staining revealed a marked reduction or loss of Nissl bodies in the same group (**Figure 3B**). Western blot analysis demonstrated that the expression levels of the tight junction (TJ) proteins ZO-1, occludin, and claudin-5 were significantly decreased in CUMS mice relative to the control group (**Figures 3C–E**). ELISA measurements indicated that the IL-6 and TNF-α levels were elevated in both the serum and the PFC of CUMS mice (**Figures 3F–I**). Treatment with LQYY or FLX mitigated the observed neuronal abnormalities, increased the expression of ZO-1, occludin, and claudin-5, and reduced the levels of IL-6 and TNF-α. Both LQYY and FLX also increased the levels of 5-HT and BDNF in the PFC (**Figures 3J, K**). In contrast, MC did not improve these outcomes.

### 3.4 LQYY alleviates CUMS-induced colonic inflammation and enhances intestinal barrier function in mice

**Figure 4:**
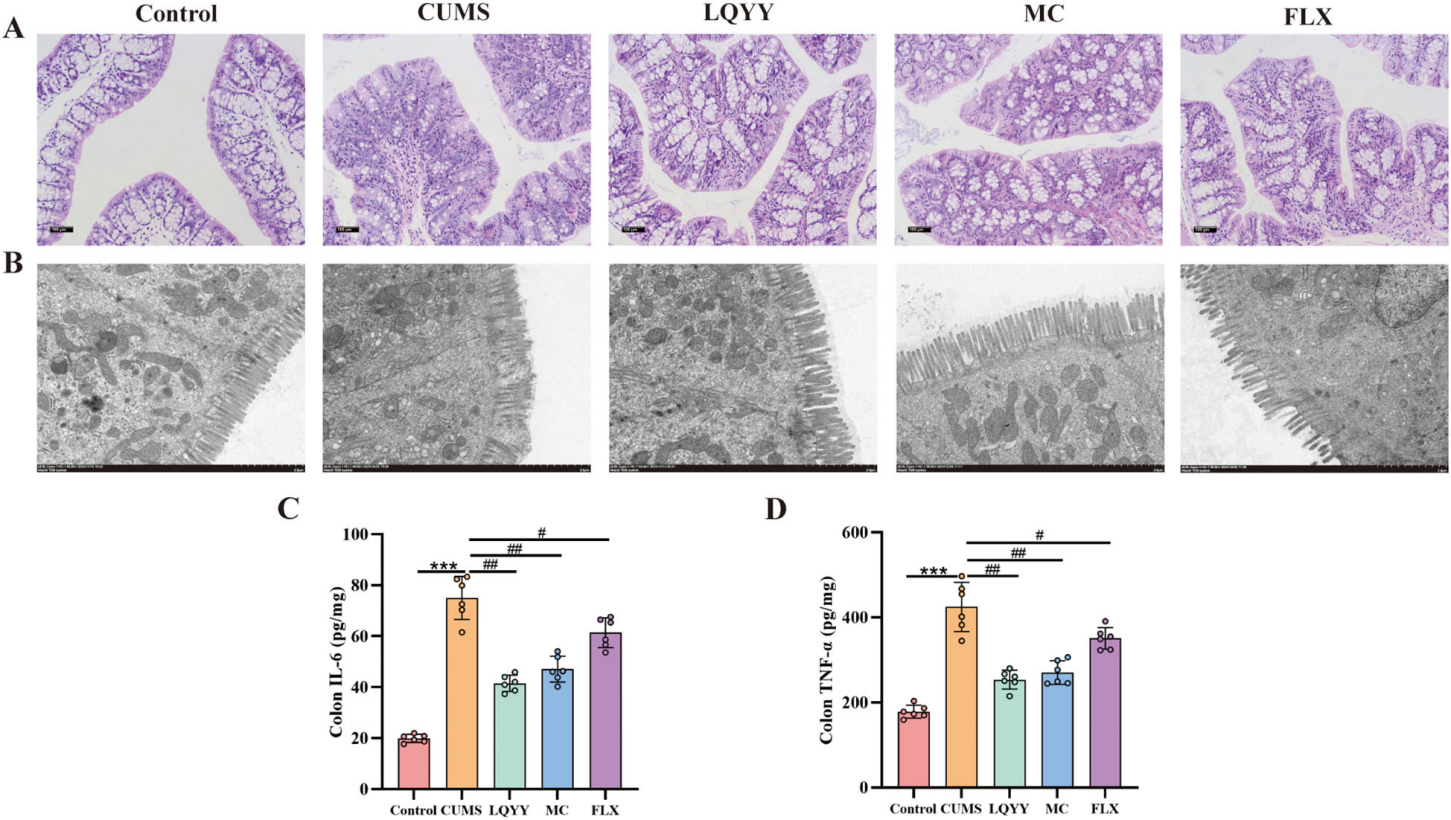
LQYY attenuates CUMS-induced colonic inflammation and restores barrier integrity. **(A)** H&E-stained colonic sections (×200, scale bar = 100 μm, n = 3). **(B)** Transmission electron microscopy (×8000, 2μm) (n = 3). **(C, D)** Colonic TNF-α and IL-6 measured by ELISA (n = 6). ***P < 0.001 *vs* control; ^###^*P* < 0.001 *vs* CUMS.

Histological analysis of colonic tissues from CUMS mice showed fewer goblet cells and marked infiltration of inflammatory cells compared to controls (**Figure 4A**). Transmission electron microscopy revealed shortened or fractured microvilli, disrupted TJ, and ultrastructural abnormalities of the epithelial barrier (**Figure 4B**). Treatment with LQYY or MC increased goblet-cell numbers, reduced inflammatory infiltration, restored microvillar density and organization, and returned TJ to near-normal appearance, whereas FLX treatment did not appreciably reverse these structural alterations. ELISA measurements indicated that the levels of IL-6 and TNF-α were elevated in the colonic tissues of CUMS mice and were significantly reduced by LQYY, MC, and FLX (**Figures 4C, D**).

### 3.5 LQYY modulates gut microbiota diversity and composition in CUMS-induced mice

**Figure 5:**
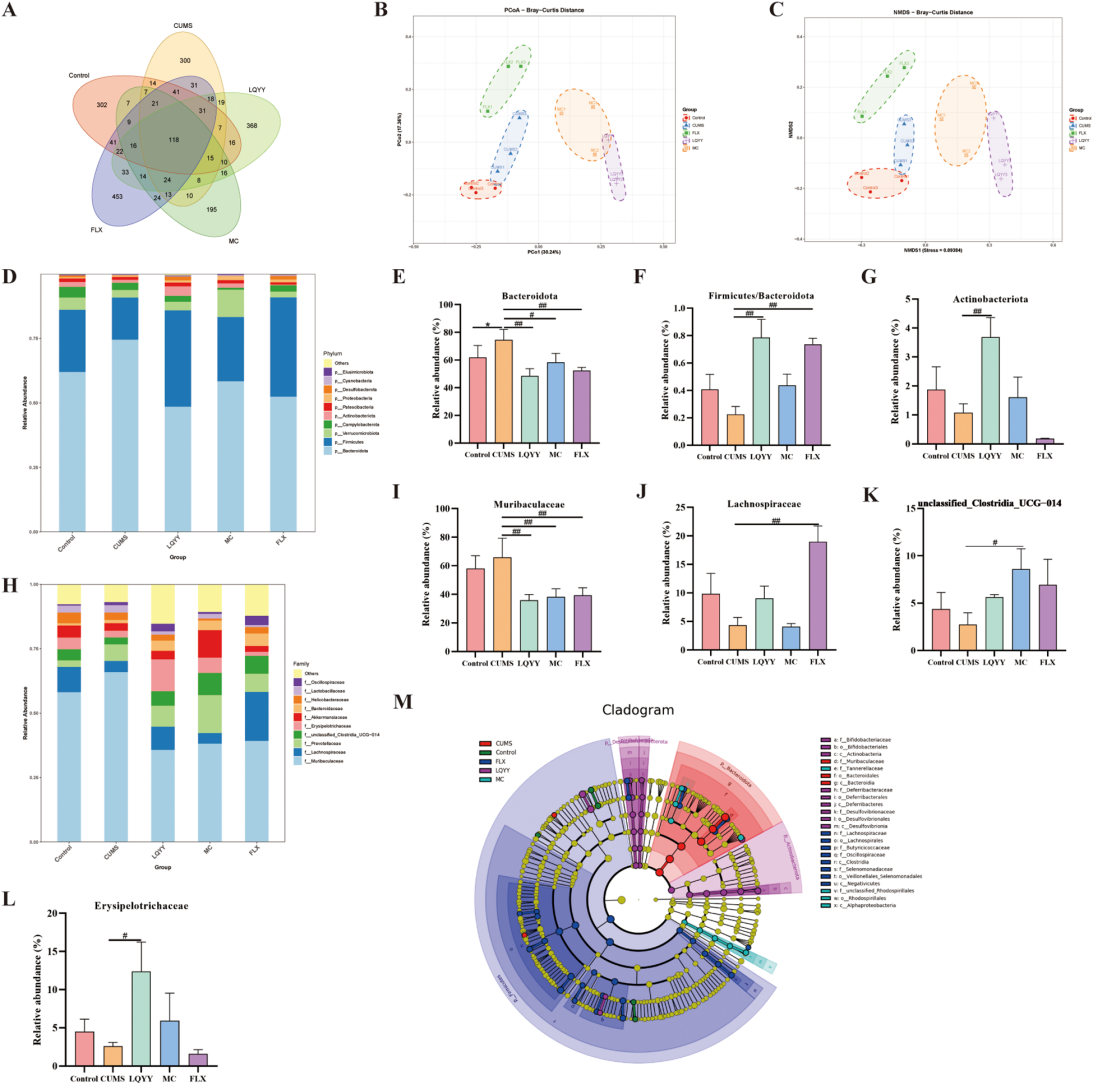
LQYY restores gut homeostasis in CUMS-induced mice. **(A)** Venn diagram of ASVs. **(B)** PCoA. **(C)** NMDS. **(D)** Phylum-level composition (stacked bar plot). **(E–G)** Phylum-level metrics: relative abundance of *Bacteroidota*, *Firmicutes/Bacteroidota* ratio, and relative abundance of *Actinobacteriota*. **(H)** Family-level composition (stacked bar plot). **(I–L)** Family-level taxa: *Muribaculaceae*, *Lachnospiraceae*, *unclassified_Clostridia_UCG-014*, and *Erysipelotrichaceae*. **(M)** LEfSe (LDA) cladogram showing differentially abundant taxa across groups. (n = 3 per group). *P < 0.05 *vs* control; ^#^*P* < 0.05, ^##^*P* < 0.01 *vs* CUMS.

Fecal microbiota was profiled by 16S rDNA gene sequencing. At the ASVs level, a Venn diagram showed distinct group-specific communities (**Figure 5A**). Alpha diversity did not differ significantly among groups (**Supplementary Figure S2**). Beta-diversity analyses (PCoA and NMDS) revealed distinct compositional separation between the CUMS group and all other groups (**Figures 5B, C**). At the phylum level, *Firmicutes*, *Bacteroidota*, and *Actinobacteriota* were the predominant taxa (**Figure 5D**). LQYY increased the *Firmicutes-*to*-Bacteroidota* ratio and *Actinobacteriota* while decreasing *Bacteroidota* (**Figures 5E–G**). At the family level, LQYY enriched *Erysipelotrichaceae* and reduced *Muribaculaceae* (**Figures 5I, L**); FLX increased *Lachnospiraceae* and decreased *Muribaculaceae* (**Figures 5H–J**); and MC increased *unclassified_Clostridia_UCG-014* while lowering *Muribaculaceae* (**Figures 5I, K**). LEfSe identified *Muribaculaceae* (family to species) and *Bacteroidota* (phylum to order) as signature taxa of CUMS-associated dysbiosis, whereas *Actinobacteriota* (phylum to class) and *Bifidobacteriaceae* (order Bifidobacteriales to species) were dominant in LQYY-treated mice (*P* < 0.05, LDA > 3.0; **Figure 5M**).

### 3.6 LQYY increases fecal ACE levels of CUMS-induced mice

**Figure 6:**
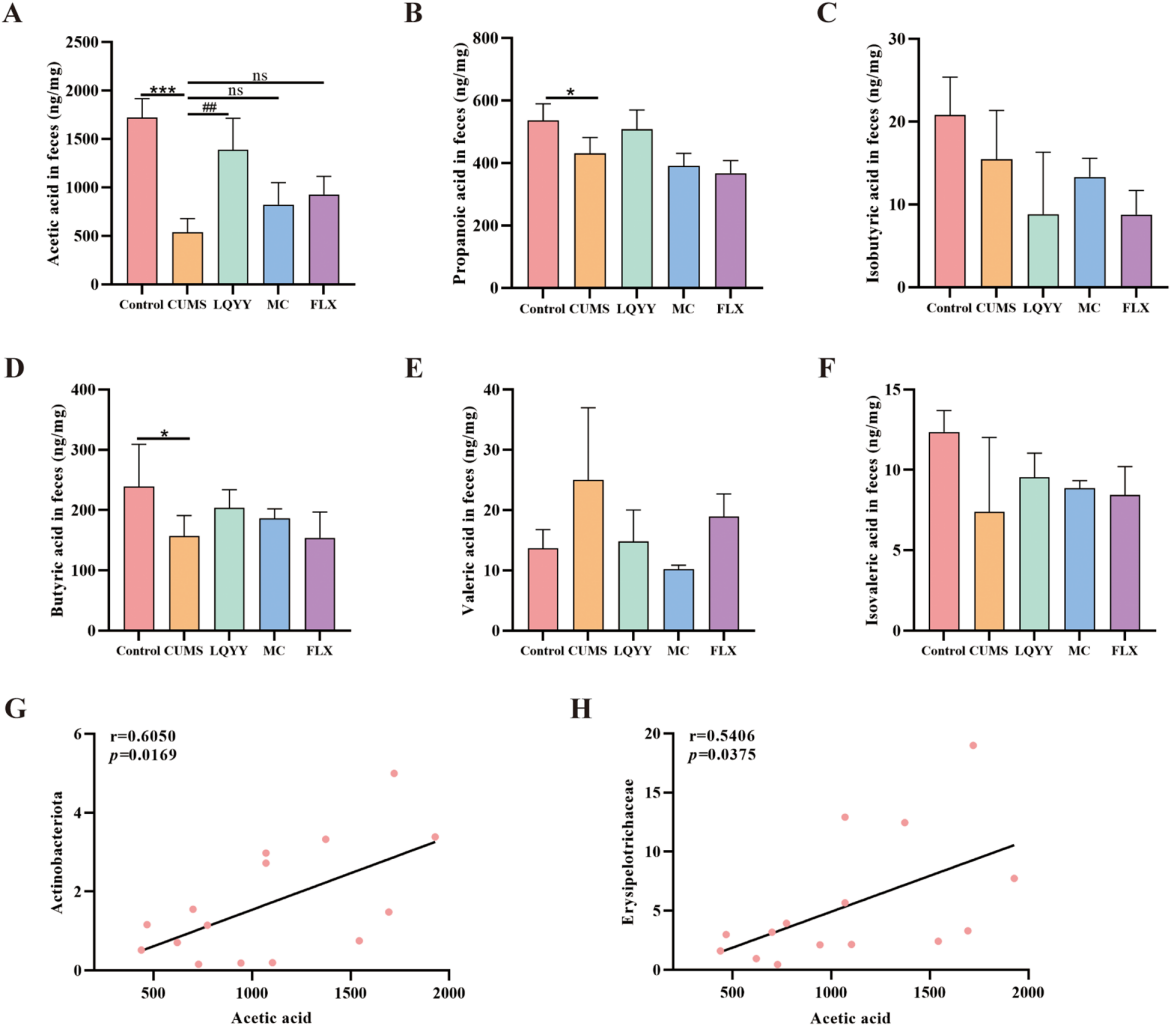
LQYY modulates fecal SCFAs in CUMS mice. **(A–F)** Fecal levels of ACE, propionate, isobutyrate, butyrate, valerate, and isovalerate. **(G)** Correlation between ACE and *Actinobacteriota*. **(H)** Correlation between ACE and *Erysipelotrichaceae*. (n = 3 per group). *P < 0.05, ***P < 0.001 *vs* control; ^##^*P* < 0.01 *vs* CUMS.

The concentrations of fecal SCFAs—including ACE, propionic acid, isobutyric acid, butyric acid, valeric acid, and isovaleric acid—were quantified (**Figures 6A–F**). The CUMS group showed reduced levels of ACE, propionic acid, and butyric acid compared to the control group, whereas treatment with LQYY significantly restored ACE levels. We then assessed associations among ACE, *Actinobacteriota*, and *Erysipelotrichaceae*—taxa that changed at the phylum and family levels in our models—and found positive correlations of acetate with both *Actinobacteriota* and *Erysipelotrichaceae* (**Figures 6G, H**).

### 3.7 ACE upregulates FFAR2 to ameliorate LPS-induced inflammation in BV-2 cells

**Figure 7:**
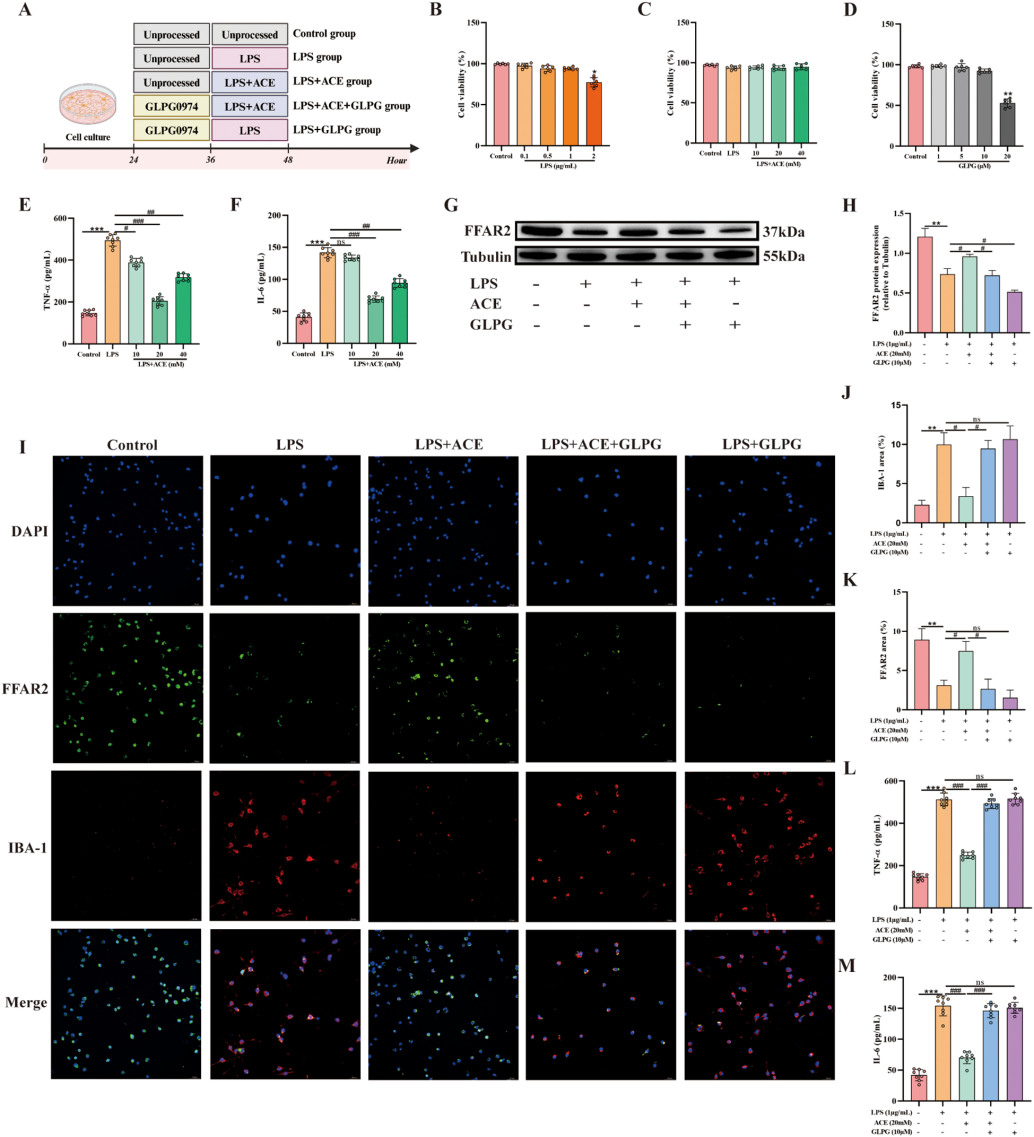
ACE suppresses microglial inflammation via FFAR2 *in vitro*. **(A)** Experimental design. **(B-D)** BV-2 viability after LPS, ACE, or the FFAR2 antagonist GLPG0974 (GLPG) (n = 6). **(E, F)** Dose-response of ACE on TNF-α and IL-6 by ELISA (n = 8). **(G, H)** FFAR2 protein by Western blot (n = 3). **(I)** Immunofluorescence of IBA-1 (red) and FFAR2 (green) (20×, scale bar, 50 μm, n = 3). **(J, K)** Quantification of IBA-1 and FFAR2 (n = 3). **(L, M)** TNF-α and IL-6 in BV-2 supernatants by ELISA (n = 8) *P < 0.05, **P < 0.01, ***P < 0.001 *vs* control; ^#^*P* < 0.05, ^##^*P* < 0.01, ^###^*P* < 0.01 *vs* LPS.

BV-2 cells were treated according to the experimental scheme outlined in **Figure 7A**. We first used CCK-8 to define working concentrations of ACE, LPS, and the FFAR2 antagonist GLPG0974; ACE showed no cytotoxicity across the tested range (**Figures 7B–D**). Subsequently, we evaluated the impact of different ACE concentrations on inflammatory factors via ELISA to establish the optimal dose (**Figures 7E, F**). In cells stimulated with LPS, co-treatment with ACE increased FFAR2 expression and reduced IBA-1, IL-6, and TNF-α compared to the LPS-only group, whereas FFAR2 blockade with GLPG0974 blunted ACE-induced FFAR2 upregulation and restored IBA-1 and pro-inflammatory cytokines (**Figures 7G–M**).

### 3.8 LQYY alleviates CUMS-induced depression and constipation in mice by relieving neuroinflammation and increasing colonic 5-HT levels via ACE/FFAR2

**Figure 8:**
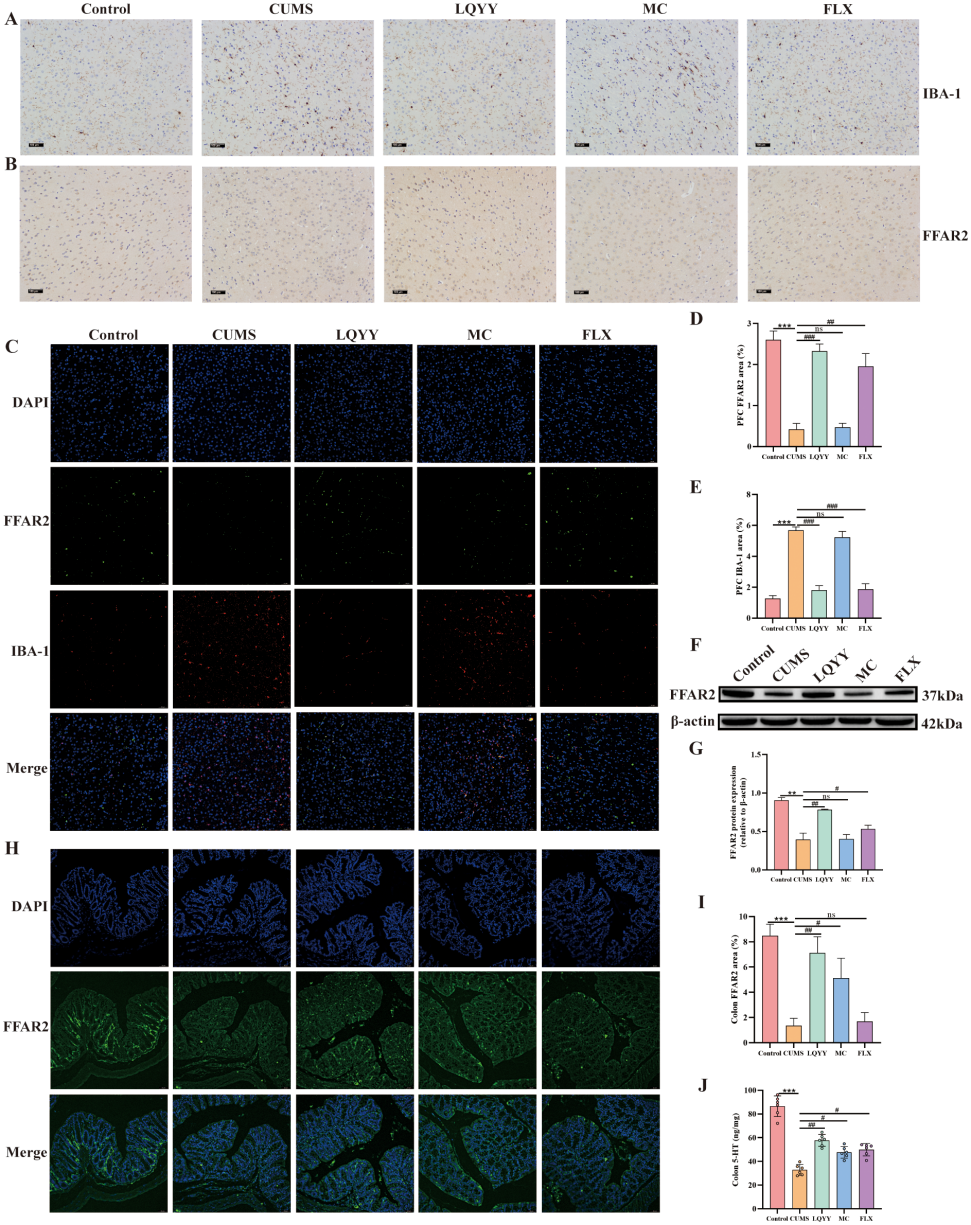
LQYY ameliorates depression- and constipation-related phenotypes via ACE/FFAR2 signaling. **(A, B)** IBA-1 and FFAR2 in PFC by immunohistochemistry (×200, scale bar, 100 μm, n = 3). **(C)** Double-label immunofluorescence for IBA-1 (red) and FFAR2 (green) in PFC (20×, scale bar, 50 μm, n = 3). **(D, E)** Quantification of IBA-1 and FFAR2 in PFC. **(F, G)** FFAR2 protein in PFC by WB (n = 3). **(H)** FFAR2 immunofluorescence in colon (20×, scale bar, 50 μm, n = 3). **(I)** Quantification of colonic FFAR2. **(J)** Colonic 5-HT measured by ELISA (n = 6). *P < 0.05, **P < 0.01, ***P < 0.001 *vs* control; ^#^*P* < 0.05, ^##^*P* < 0.01 *vs* CUMS.

The expression of IBA-1 and FFAR2 in the PFC, as well as FFAR2 and 5-HT in the colon, was quantified. In the PFC, immunohistochemistry showed reduced IBA-1 and increased FFAR2 in the LQYY and FLX groups versus CUMS (**Figures 8A, B**). Double-label immunofluorescence localized FFAR2 to microglia; The fluorescence intensity analysis revealed that CUMS decreased FFAR2 and increased IBA-1, while LQYY and FLX treatment reversed these changes (**Figures 8C–E**). WB analysis corroborated the upregulation of FFAR2 protein in the PFC following LQYY and FLX treatment (**Figures 8F, G**). In the colon, LQYY and MC treatment increased FFAR2 expression and 5-HT levels relative to CUMS mice (**Figures 8H–J**).

## 4 Discussion

**Figure 9:**
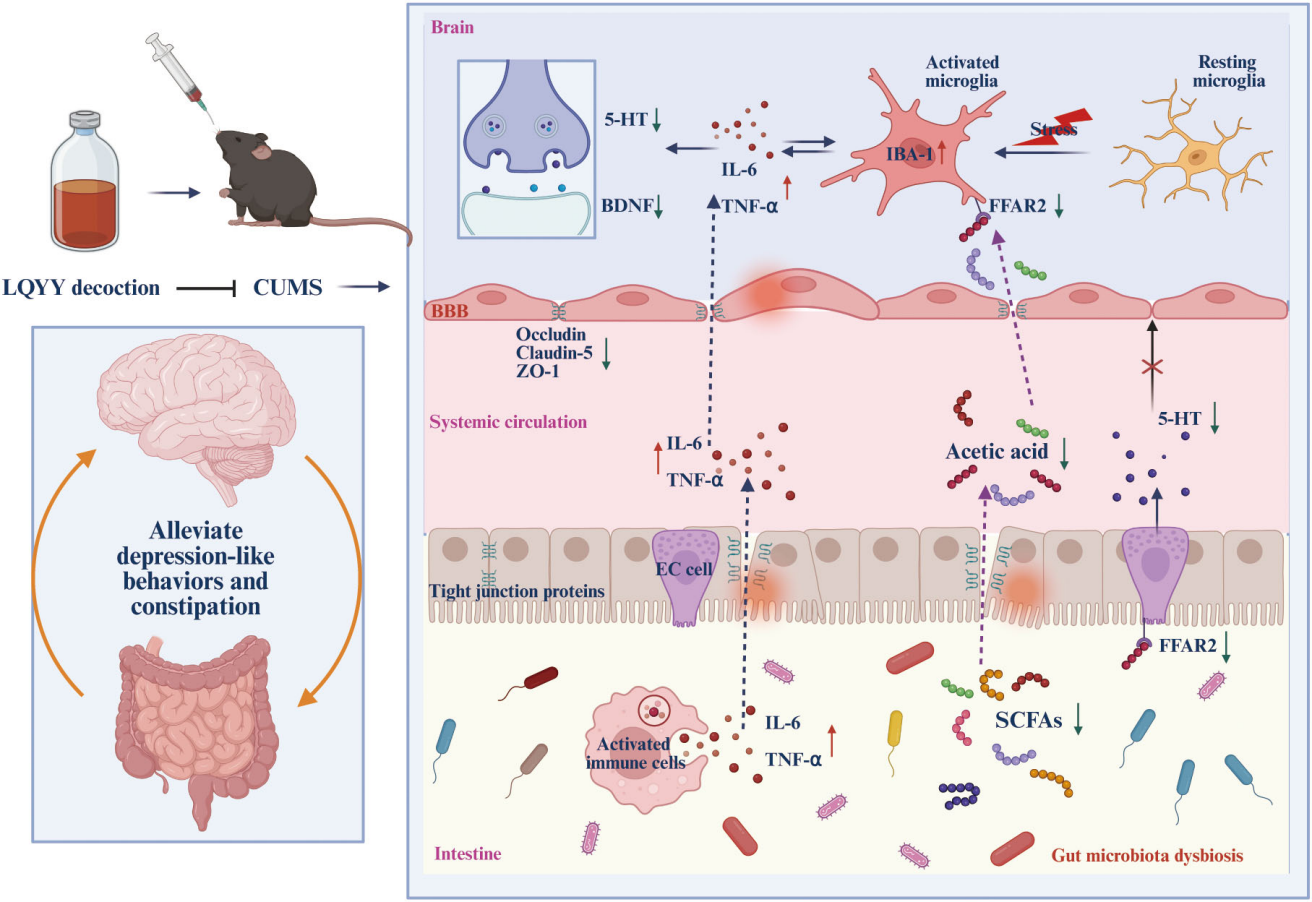
The schematic diagram of LQYY treating CUMS-induced depression with constipation via ACE/FFAR2 of microbiota-gut-brain axis. LQYY supplementation can regulate the composition and metabolism of gut microbiota (1), thereby, contribute to gut barrier function and reducinginal inflammation (2), increasing 5-HT release by activating FFAR2 receptors on ECs through the metabolite ACE to promote intestinal motility (3); Moreover, on the one hand, ACE protects the integrity of the BBB, inhibiting the transmission of inflammatory signals between the intestine, periphery and brain (4), on the other hand, ACE crosses the BBB, activating the FFAR2 receptor on microglia, inhibiting the activation and inflammation of microglia in the CNS (5). Therefore, the supplement of LQYY beneficially impacts on depression and constipation, via restoring gut microbiota, increasing the metabolite ACE and its regulatory role in the gut-brain axis.

In our research, we evaluated the effects of LQYY on CUMS-induced depression with constipation in mice. Overall, our findings indicate that LQYY treatment has the potential to improve behavioral changes and intestinal transit disorders in mice. This improvement appear to be achieved by reestablishing the disrupted gut microbiota structure, increasing the level of intestinal metabolites ACE, activating FFAR2 receptors, thereby inhibiting neuroinflammation and enhancing colonic 5-HT secretion. Our study demonstrates that CUMS induction disrupts the integrity of the colonic epithelial barrier by damaging colonic microvilli and TJ proteins. Furthermore, CUMS elevates BBB permeability by diminishing the expression of ZO-1, claudin-5, and occludin. LQYY can partially restore its damage and expression. Notably, the positive control drugs FLX and MC exhibited protective effects specifically in the PFC and colon, respectively. This finding highlights a potential therapeutic advantage of LQYY, which demonstrates broader protective effects across both systems. Furthermore, the improvement by LQYY was rendered ineffective by using broad-spectrum antibiotics, which further emphasized the significant role the intestinal microbiota in regulating behavioral changes and intestinal transit function. In conclusion, LQYY supplementation is believed to positively affect depression and constipation by restoring gut microbiota, increasing the metabolite ACE and its role in regulating the gut-brain axis. These results are summarized in **Figure 9**. This suggests that upregulation of the ACE/FFAR2 pathway is a key mechanism for targeting microbes in the treatment of depression accompanied by constipation.

In recent years, growing evidence has shown that the intestinal microbiota is closely linked to depression and constipation (Margolis et al., 2021). Therefore, modulation of gut microbiota is considered a promising therapeutic strategy for depression accompanied by constipation. In this study, LQYY was used as a potential therapeutic agent to investigate the role of intestinal microbiota in treating depression and constipation. The curative effect of LQYY on depression with constipation was confirmed by using behavioral tests such as SPT, OFT, FST, NSFT, as well as intestinal transit function tests such as the fecal water content, the time of the first black stool, and intestinal propulsion rate. Furthermore, we used ABX treatment to explore whether LQYY improved depressive and constipation symptoms through the intestinal microbiota. Mice were given free access to drinking water containing multiple antibiotics for 7 days. This one week antibiotic regimen was chosen based on prior evidence showing that such short-term intervention does not induce behavioral changes in mice (Hao et al., 2024). Consistent with this, our findings further confirmed that 1-week of oral antibiotic administration had no effect on the behavior of mice (**Supplementary Figure S3**). After administration of ABX to clear the gut microbiota, the antidepressant and anticonstipation effects of LQYY were blunted, indicating that gut microbiota is essential for LQYY to exert its therapeutic effects.

After confirming that LQYY improved behavioral changes and intestinal motility disorders in a microbiota-dependent manner, we conducted further investigations to examine specific alterations in the gut microbiota and microbial metabolite composition. Previous studies have show that compared with healthy controls, patients with major depressive disorder (MDD) exhibit a notable increase in the abundance of the phylum Bacteroidota and a marked decrease in the phylum Firmicutes (Jiang et al., 2015). Additionally, a similar phenomenon was also reported in the study of elderly patients with functional constipation (Guo et al., 2020) consistent with our results, LQYY treatment could restore the ratio of Firmicutes/Bacteriodota (F/B). In addition, the researchers have found that phylum Actinobacteriota and class Actinobacteria, along with the family Bifidobacteriaceae and genus Bifidobacterium, exerted a protective effect against MDD (Chen et al., 2022; Zhao et al., 2024). Similarly, in our research, LQYY increased the abundance of Actinobacteriota and Bifidobacteriaceae. Moreover, Jeong et al. found that the levels of the family Erysipelotrichaceae were markedly decreased in loperamide-induced constipation rats versus the normal group, while the levels of the family Erysipelotrichaceae in the fecal samples of the probiotic-treated groups were boosted (Jeong et al., 2023). We also found that LQYY treatment could increase the abundance of the family Erysipelotrichaceae in CUMS-induced depression with constipation. In addition, we found that LQYY can increase the content of SCFAs, especially ACE, and Erysipelotrichaceae and Actinobacteriota were all positively correlated with ACE. Unlike LQYY, MC and FLX influence the gut microbiota by focusing on distinct microbial populations. Overall, these findings suggest that LQYY enhances the composition of intestinal microbiota, particularly by promoting Actinobacteriota, Erysipelotrichaceae, and Bifidobacteriaceae, and inhibiting Bacteroidota. This modulation may contribute to preventing behavioral changes and accompanying constipation in CUMS-induced depression mice.

Gut microbiota imbalance may contribute to intestinal inflammation, which plays a critical role in disrupting the intestinal mucosal barrier (Li et al., 2022b). The BBB dysfunction has been associated with depression, stress, neuroinflammation, and the imbalance of CNS homeostasis (Medina-Rodriguez and Beurel, 2022). Recent research has shown that BBB disruption in CUMS-induced mice is linked to reduced levels of ZO-1, claudin-5, and occludin (Shi et al., 2024). Elevated intestinal permeability, along with BBB disruption, facilitates the transmission of inflammatory signals between the gut and brain, aligning with our observations. Our study found that CUMS mice exhibited pathological changes in the colon, including a decrease in goblet cells and extensive inflammatory cell infiltration, destruction of colonic microvilli and TJ proteins, increased expression of TNF-α and IL-6 in the colon and serum, and significantly reduced expression of ZO-1, occludin, and claudin-5 in the PFC. LQYY can improve all of the above changes.

As stated above, firstly, the imbalance of intestinal flora can facilitate the entry of peripheral inflammatory factors into the brain, contributing to the development of CNS inflammation. Secondly, when responding to stress, resting microglia are transformed into activated microglia, releasing pro-inflammatory cytokines such as IL-6 and TNF-α, thereby exacerbating the neuroinflammatory response (Wu et al., 2021; Xian et al., 2022). Highly activated microglia cells in the brain are considered a marker of neuroinflammation, and ionized calcium-binding adaptor molecule 1 (IBA-1) is widely recognized as a marker of microglial activation (Li et al., 2021). 5-HT, a key neurotransmitter, also serves as a crucial regulator of the gut-brain axis. Approximately 5% of 5-HT is synthesized in the CNS, and it is highly concentrated in the cerebral cortex and nerve synapses, when its level decreases, it can cause neuronal conduction dysfunction in the brain thus triggering depression (Okaty et al., 2019). The first-line antidepressant drugs are based on blocking the reuptake of 5-HT, thus increasing its in the synaptic cleft, to achieve an antidepressant effect (Vahid-Ansari and Albert, 2021). Tryptophan (Trp) metabolism has three routes: 5-HT, kynurenine (Kyn) and indole. When the organism is in a state of inflammation or stress, the activity of the important rate-limiting enzyme in the Kyn metabolism pathway, indoleamine 2, 3-dioxygenase (IDO) increases, which promotes the metabolism of Trp to Kyn, resulting in insufficient content of Trp, a precursor substance for the synthesis of 5-HT in the brain, affecting the production of 5-HT and thereby exacerbating depression (Zhang et al., 2020). In this study, we found that CUMS induced neuroinflammation and decreased the levels of neurotransmitters, characterized by the overexpression of IBA-1, elevated levels of the pro-inflammatory cytokines IL-6, TNF-α and a decrease in 5-HT levels in the PFC. LQYY treatment effectively reduced the expression of IBA-1, IL-6, TNF-α and increased the expression of 5-HT. BDNF is the most plentiful and ubiquitous neurotrophic factor in the CNS. Studies have shown that in animals, depressive patients, and samples of depressive patients after death, a decrease in brain BDNF levels was found (Song et al., 2020). In addition, microglia activation can reduce BDNF secretion, inhibiting neurodevelopment, growth, maturation, impairing synaptic plasticity, and ultimately disrupting neurons to induce depression (Prowse and Hayley, 2021). In this study, we found that in the CUMS, H&E and Nissl staining showed neuronal damage, the levels of BDNF were significantly decreased, while LQYY improved neuronal damage and increased the levels of BDNF in the PFC.

Growing evidence indicates that SCFAs serve not only as an energy source but also as natural ligands for FFARs, the major FFARs activated by SCFAs are FFAR3, FFAR2, and GPR109A (Ikeda et al., 2022). FFAR2 recognizes a wide range of SCFAs, including ACE, propionic acid, butyric acid, and caproic acid, with ACE being the most selective (Tan et al., 2014). Research shows that gut microbiota dysbiosis with reduced SCFAs is linked to suppressed FFAR2 signaling, which is known to regulate various biological pathways involved in energy metabolism, intestinal cellular homeostasis, gut motility, and inflammatory response (Mishra et al., 2020). 5-HT plays a crucial role in initiating gastrointestinal peristalsis and secretory reflexes, regulating normal autonomic defecation and visceral sensation (Liu et al., 2021). 5-HT produced in intestinal EC cells can accelerate intestinal peristalsis and treat constipation (Bai et al., 2022). The secretion of 5-HT from EC cells has been shown to be facilitated by increasing FFAR2 expression (Iwasaki et al., 2019). These results suggest that the gut microbiota profile and SCFAs levels are closely linked to 5-HT signaling. Consistent with this idea, LQYY treatment could replenish the loss of 5-HT, FFAR2, and ACE in CUMS mice. Moreover, Erny et al. (2015) found that mice with a defective FFAR2 gene showed severe abnormalities in microglia, with increased dendritic length, segment number, branching points, and cell volume, suggesting that FFAR2 directs the maturation and function of microglia. In their study on the protective effects of ACE against postoperative neurocognitive disorders in aged mice, Wen et al. (2020) demonstrated that ACE inhibits microglial activation and reduces neuroinflammation by increasing FFAR2 expression. This effect was attenuated when FFAR2 expression on BV-2 cells was silenced by siRNA. Similarly, our findings show that ACE can activate FFAR2 in LPS-treated BV-2 cells, inhibit the expression of IBA-1, TNF-α and IL-6, thereby inhibiting neuroinflammation, and this effect is impaired by FFAR2 inhibitors (GLPG0974). Besides, we have shown that LQYY increased the content of ACE levels in CUMS-induced depression with constipation mice, and to further verify the effect of LQYY on FFAR2 signaling *in vivo*, we detected the levels of FFAR2, IBA-1, IL-6 and TNF-α, and found that LQYY increased the expression of FFAR2 and decreased the expression of IBA-1, IL-6 and TNF-α. In summary, we suggest that LQYY may achieve the purpose of treating depression and constipation by increasing ACE and FFAR2, inhibiting neuroinflammation and increasing colonic 5-HT expression.

Our findings demonstrate that the CUMS procedure successfully induces a composite phenotype of behavioral changes and constipation in mice. This model effectively recapitulates the clinical presentation of a specific subpopulation of patients—those suffering from depression with co-morbid constipation. The parallel suggests that shared underlying pathophysiological mechanisms, likely involving chronic stress-induced malfunctions of the gut-brain axis, can simultaneously drive both affective and gastrointestinal symptoms. In the clinical context, constipation can be attributed to at least two factors: the intrinsic pathophysiological link between depression and gut dysfunction, and the extrinsic factor of side effects from certain antidepressant medications, such as tricyclics and some selective serotonin reuptake inhibitors (SSRIs). Therefore, CUMS model specifically elucidates the first mechanism, offering a valuable preclinical tool to investigate the neurobiological and gastrointestinal underpinnings of depression-associated constipation that is intrinsic to the disease state itself, paving the way for developing therapies that target the core pathophysiology rather than merely managing drug side effects.

Nevertheless, the experiment has several limitations. First, we used only young male mice of a single strain, while crucial for minimizing variability and establishing a clear mechanistic foundation, does not fully recapitulate the clinical demographics of depression-constipation comorbidity, which shows a higher prevalence in females and aging populations. Future study employing female, aged, or genetically diverse animal models to better reflect clinical heterogeneity. Second, LQYY, as a traditional herbal formulation, contains numerous active compounds. While our findings highlight its therapeutic effects and mechanisms, the role of specific active ingredients remain unidentified. Future research should focus on comparing the efficacy of key active components, which is crucial for a comprehensive of its multi-component synergistic effects. Third, while LQYY can improve *Actinobacteriota* and *Erysipelrichaceae*, we did not demonstrate whether its action is by reshaping the gut microbiota structure to modulate the abundance or directly promote the growth of these bacteria. Future study can mono-colonization with *Actinobacteriota* or *Erysipelotrichaceae* alone to explore its effects on anti-depression and anti-constipation.

## 5 Conclusion

In conclusion, our findings suggest that LQYY alleviates CUMS-induced behavioral changes and constipation, potentially through mechanisms involving the upregulation of FFAR2 expression, suppression of CNS inflammation, and enhancement of colonic 5-HT synthesis. These effects may be attributed to its positive impact on preserving the integrity of both the intestinal barrier and the BBB, modulating the structure of the gut microbiota and metabolite ACE, and influencing gut-brain interactions. These findings offer fresh perspectives on intestinal microbiota-mediated improvement of depression with constipation and could foster advancements in treatment and prevention approaches for depression and other gastrointestinal disorders. Additionally, our results support the potential of LQYY as an effective intervention for the treatment of depression accompanied by constipation.

## Data Availability

The original contributions presented in the study are included in the article/**Supplementary Material**. Further inquiries can be directed to the corresponding authors.
