# Supplementary material for: Liqi Yangyin formula ameliorates CUMS-induced depression and comorbid constipation via ACE/FFAR2 modulation of the microbiota-gut-brain axis
Source: Front Cell Infect Microbiol. 2025 Nov 7;15:1692110. doi: 10.3389/fcimb.2025.1692110 (PMC12634565; doi:10.3389/fcimb.2025.1692110)
Supplement: Supplementary file 1 [file Table1.docx]

**Supplementary Materials**

**Additional figures**


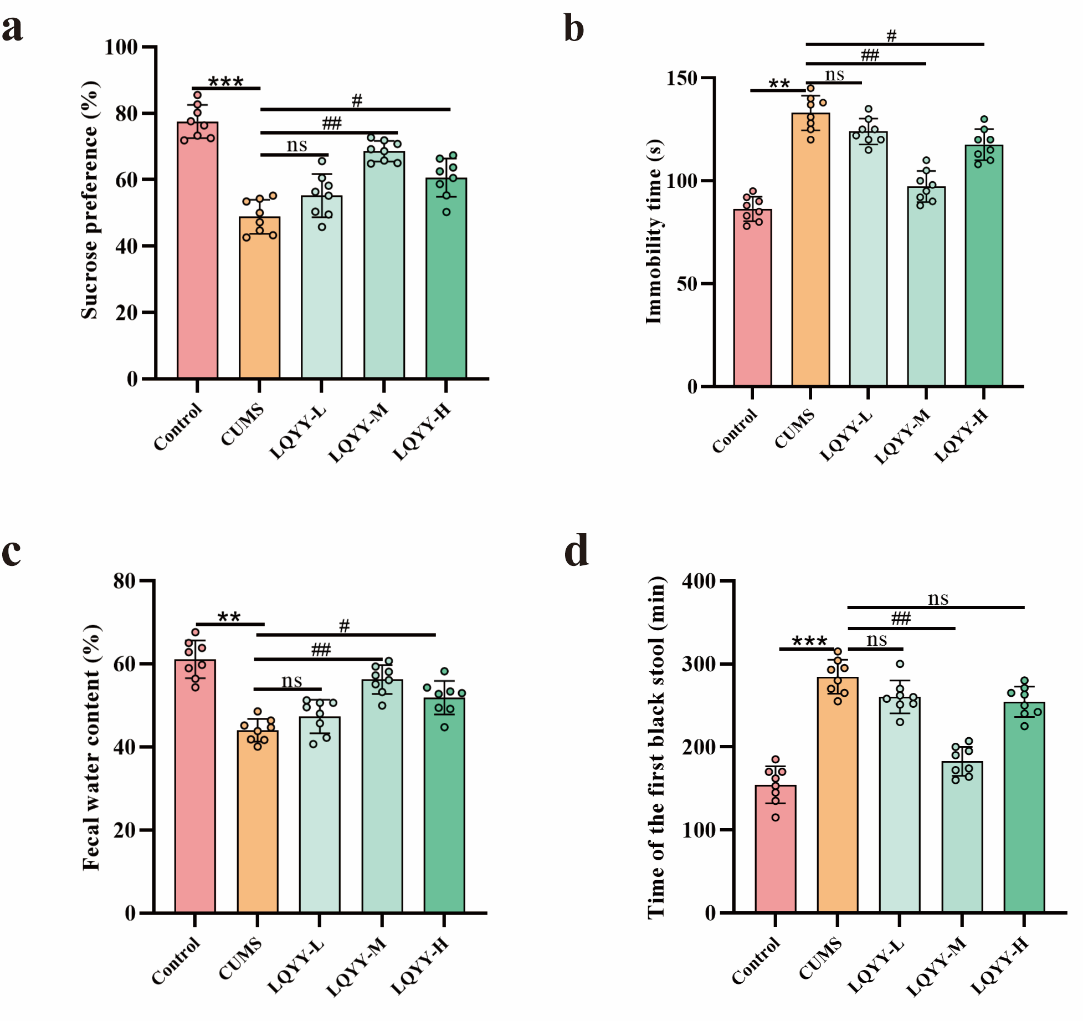


**Fig. S1** The effect of different doses of LQYY on CUMS-induced depression with constipation in mice. (a) Sucrose preference. (b) The immobility time in the FST. (c) Fecal water content. (d) Time of the first black stool. Data represent the mean ± SD (n=8 per group). ***P*<0.01 and ****P*<0.001, when compared to the control; ^#^*P*<0.05 and ^##^*P*<0.01, when compared to the CUMS. LQYY-L, Low dose group; LQYY-M, Middle dose group; LQYY-H, High dose group.


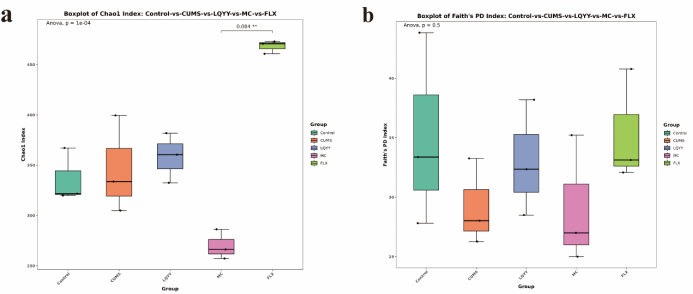


**Fig. S2** Comparison of alpha diversity of gut microbiota in mice with different treatment groups. **(a)** Chao1 index. **(b)** Faith’s PD index. (n=3 per group). No significant differences between groups.

Seven-week-old SPF male C57BL/6 mice were maintained in controlled environmental conditions (23 ± 2 °C, 60% humidity, and 12/12 h light/dark cycles). After one week of acclimation, mice were randomly divided into two groups: Control, Control+ABX. The treatments were as follows: (1) Control group: served as a negative control; (2) Control+ABX group: accepted an antibiotic cocktail consisting of ampicillin (1 g/L; A830931; Macklin), vancomycin (500 mg/L; V820413; Macklin), neomycin sulfate (1 g/L; N8090; Solarbio) and metronidazole (1 g/L; M813526; Macklin) in the mice’s daily drinking water for one-week. Through the SPT, FST, NSFT, OFT, fecal water content and time of the first black stool tests, we observed that one week of ABX intervention had no effect on the behavior of mice (Fig. 1). Our findings suggest that a one-week antibiotic treatment does not impact the behaviors of SPF mice.


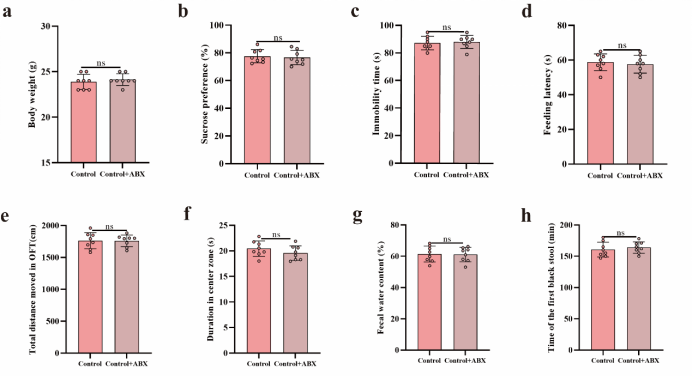


**Fig. S3** The 1-week ABX intervention did not significantly impact the behaviors of SPF mice. (a) Body weight. (b) Sucrose preference. (c) The immobility time in the FST. (d) The feeding latency time in NSFT. (e) The total distance traveled in OFT. (f) The duration in center zone in OFT. (g) Fecal water content. (h) Time of the first black stool. Data represent the mean ± SD (n=8 per group). No significant difference was found between control and control + ABX group.
